# Renoprotective effect of N-acetylcysteine depends upon the severity of the ischemia reperfusion injury

**DOI:** 10.1590/1414-431X2021e9941

**Published:** 2021-09-03

**Authors:** M. Watanabe, F.T. Borges, E.A. Pessoa, C.D. Fonseca, S.M. Fernandes, R.C. Drew, R.A. Volpini, M.F.F. Vattimo

**Affiliations:** 1 Laboratório Experimental de Modelos Animais, Escola de Enfermagem, Universidade de São Paulo, São Paulo, SP, Brasil; 2 Divisão de Nefrologia, Universidade Federal de São Paulo, São Paulo, SP, Brasil; 3 Escola Paulista de Enfermagem, Universidade Federal de São Paulo, São Paulo, SP, Brasil; 4 Department of Exercise and Health Sciences, College of Nursing and Health Sciences, University of Massachusetts Boston, Boston, MA, USA; 5 Departamento de Nefrologia, Faculdade de Medicina, Universidade de São Paulo, São Paulo, SP, Brasil

**Keywords:** Acute kidney injury, Oxidative stress, Ischemia-reperfusion

## Abstract

Acute kidney injury (AKI) is a common complication in seriously ill patients, while renal ischemia-reperfusion (I/R) injury is the most frequent event in this oxidative renal injury. N-acetylcysteine (NAC) is a small molecule containing a thiol group that has antioxidant properties, promoting detoxification and acting directly as a free radical scavenger. In this study, the protective effect of NAC was investigated in short-term (30 min) and long-term (45 min) ischemic AKI. This was achieved via clamping of the renal artery for 30 or 45 min in Wistar rats to induce I/R injury. AKI worsened with a longer period of ischemia (45 compared to 30 min) due to probable irreversible damage. Preconditioning with NAC in short-term ischemia improved renal blood flow and increased creatinine clearance by reducing oxidative metabolites and increasing antioxidant capacity. Otherwise, NAC did not change these parameters in the long-term ischemia. Therefore, this study demonstrated that the period of ischemia determines the severity of the AKI, and NAC presented antioxidant effects in short-term ischemia but not in long-term ischemia, confirming that there is a possible therapeutic window for its renoprotective effect.

## Introduction

Acute kidney injury (AKI) is a common complication that affects 5-10% of critically ill patients. It is associated with increased mortality, morbidity, and high hospital costs (1). In the hospital setting, ischemic kidney injury is the most frequent event, mostly due to transplantation surgery, prolonged resuscitation, shock, and sepsis, and in these situations it is characterized as renal ischemia-reperfusion (I/R) injury (1,2). Ischemia is described as the impairment of blood and nutrient supply to renal cells, causing tissue hypoxia and cellular injury (1,2). Restoration of blood flow minimizes the hypoxic insult, but exaggerated reoxygenation aggravates tissue injury by intense inflammatory response, which includes the production of reactive oxygen species (ROS), reactive nitrogen species (RNS), superoxide anions (O_2_^-^), and hydroxyl radical (OH) (2-6). Both ROS and RNS reduce antioxidant capacity and cause injury to renal cells by oxidation of proteins, peroxidation of lipids, and DNA damage. This imbalance results in cell dysfunction that, in severe settings, can progress to cell death by apoptosis or necrosis (7,8).

Several strategies have been proposed to deal with I/R induced AKI. These include antioxidant agents and antioxidant enzymes, carbon monoxide-releasing molecules, and n-acetylcysteine (NAC) (9). NAC is a water-soluble molecule and a cytosol-specific antioxidant that acts by increasing intracellular glutathione levels (GSH) and scavenging reactive species (9,10). NAC has shown antioxidant and vasodilator effects in renal vascular cells in pathological settings (10), including toxicity (11) and I/R renal injury (12). Several experimental models of AKI have successfully reported the antioxidant effect of NAC (13,14).

On the other hand, studies have failed to provide clarity regarding the interventional use of NAC, once no benefit was manifested in some conditions and even worsened outcomes were observed in severe pathology settings (13,14). Therefore, we hypothesized that the effectiveness of NAC treatment depends on the severity of the renal I/R injury. This paper evaluated whether the renoprotective effect of NAC was associated with the time *vs* severity of the renal ischemic injury.

## Material and Methods

### Animals

Adult male Wistar rats (weighing 293±6 g) were housed in a room at a controlled temperature (25°C/77°F) on alternating light/dark cycles and had free access to water and rat chow (Nuvilab CR-1; Nuvital, Brazil). The animals were obtained from the Animal Care Facility at the University of São Paulo, School of Medicine. The study was carried out in accordance with international standards for the manipulation and care of laboratory animals. The protocol was approved by the Ethical Committee of Experimental Animals, University of Sao Paulo (CEEA - protocol No. 156/09).

### Ischemia-reperfusion **injury**

The study consisted of two experimental models of renal I/R injury. Animals were anesthetized with 10 mg/kg xylazine and 90 mg/kg ketamine. Bilateral renal pedicles were exposed after ventral incision, and then the renal arteries of both kidneys were isolated and clamped for 30 or 45 min to induce ischemia. After that, the clamps were removed and reperfusion was visualized by blood flow and restoration of color in the renal tissue. Muscles and skin were sutured at the end.

### Experimental groups

SHAM (n=7; control group): an abdominal incision was made without ischemia procedures. SHAM + NAC (n=7) control group and 150 mg/kg of NAC was given intraperitoneally 60 min before abdomen incision and 10 min after the surgical procedure (15). ISC 30 (n=6; short-term ischemia): after the abdominal incision, renal arteries were clamped for 30 min. ISC 30 + NAC (n=6): short-term ischemia and 150 mg/kg of NAC was given intraperitoneally at 60 min before incision and 10 min after the surgery. ISC 45 (n=6; long-term ischemia): after the abdominal incision, renal arteries were clamped for 45 min. ISC 45 + NAC (n=6): long-term ischemia and 150 mg/kg of NAC was given intraperitoneally at 60 min before incision and 10 min after surgery.

### Procedures and timing

#### Metabolic cages for collection of urine

Short- and long-term renal ischemia were followed by 24-h reperfusion. Rats were placed into metabolic cages for measurement of 24-h urinary volume and collection of urine samples.

#### MAP and RBF measurement

Animals were anesthetized as described above after being removed from the individual metabolic cages. A polyethylene tube (PE-50) was inserted into the right carotid artery for monitoring mean arterial blood pressure (MAP) and heart rate (HR) by an electronic transducer connected to a data acquisition program (TSD104A, BIOPAC Systems Inc., USA). The renal artery was isolated after exposing the left renal pedicle, and a suitable probe was placed around it for measurement of renal blood flow (RBF), which was performed by an ultrasonic flowmeter (T402, Transonic Systems Inc., USA).

#### Collection of blood sample

After obtaining parameters of renal hemodynamics, a blood sample was collected through puncture of the abdominal aorta. Animals were submitted to euthanasia at the end of the experiment, according to guidelines for animal experimentation.

#### Tissue sample collection/preparation

The right kidney was removed, immediately cooled, and stored at -70°C. The left kidney was fixed by 10% formaldehyde solution, dehydrated by alcohol, embedded in paraffin, sectioned at 5 µm, and stained with hematoxylin and eosin.

### Renal function measurement

#### Creatinine clearance

Serum and urinary creatinine (Cr) were measured using the Jaffe method. The calculated clearance was adjusted by 100 g/rat weight (16).

#### Fractional excretion of sodium (FENa)

Serum and urinary Na were measured using flame photometry and calculated with the formula: urinary excretion = [(urinary Na × serum Cr) / (urinary Cr × serum Na)] × 100 (16).

### Renal vascular resistance

Renal vascular resistance (RVR) was calculated with the usual formula: RVR = MAP / RBF (17).

### ROS and RNS metabolites assays

#### Determination of urinary peroxides

Urinary peroxides were determined by the method of ferrous oxidation of xylenol orange version 2 (FOX-2). Xylenol orange (Sigma, USA) shows a high selectivity for the Fe^3+^ ion, producing a blue-purple complex (a = 4.3×10^4^ M^-1^cm^-1^). The values are reported as nmol/g urinary Cr (18).

#### Measurement of thiobarbituric acid reactive substances (TBARS)

Urine samples were added to 17.5% trichloroacetic acid (TCA) and 0.6% thiobarbituric acid. This mixture was heated up to 95°C in a double boiler for 20 min. The solution was removed from the double boiler and cooled in ice, followed by the addition of 70% TCA. The solution was then incubated for 20 min at room temperature and absorbance was read at 534 nm (a = 1.56 × 10^5^ M^-1^cm^-1^). The amount of TBARS is reported as nmol/g urinary Cr (19).

#### Determination of urine nitrite excretion

Urinary excretion of nitrite was measured using the Griess reaction method. A solution of 1% sulfanilamide in 5% H_3_PO_4_ containing 0.1% naphthylethylenediamine was added to aliquots of media and absorbance was read at 546 nm. Results are reported as nmol/g urinary Cr (20).

### Antioxidant activities assay

#### Analysis of soluble non-protein thiols in renal tissue

The renal tissue was homogenized in a solution containing sodium acetate, 0.5% Tween 20, and DTPA with a pH of 6.5. One aliquot was reserved for the measurement of total protein, and a second aliquot was precipitated with 10% TCA for the measurement of total thiols. After 10 min at room temperature, the quantity of thiols was determined as the mean absorbance at 412 nm (a = 13.6 × 10^3^ M^-1^cm^-1^). The amount of soluble thiols was corrected by the amount of total protein, and is reported as nmol/mg total protein (21).

#### Measurement of glutathione peroxidase activity in renal tissue

The renal tissue was homogenized with 50 mM tris-HCl buffer at a pH of 7.6 (1:5 w/v ratio). The mixture was then centrifuged (4500 *g* for 30 min at 4°C), and the supernatant was filtered and centrifuged again under the same conditions. Glutathione peroxidase was assayed using 0.15 M phosphate buffer (pH 7.0), containing 5 mM EDTA, 0.0084 M NADPH, 4 µg of GSSG-reductase, 1.125 M NaN_3_ (sodium azide), and 0.15 M GSH in a total volume of 0.3 mL. The absorbance of the samples was measured at 340 nm using a spectrophotometer, and the measurements were taken every 15 s for 300 s. The GSH-Px enzymatic activity is reported as U/g of renal tissue (22).

### Histological analysis

All sections were evaluated in a blind manner for qualitative and quantitative histological analyses.

#### Fractional interstitial area (FIA) of the renal cortex

Interstitial areas were determined by computerized morphometry (Axioskop 40-Carl Zeiss, Germany) in all experimental groups. In each renal cortex section, 20 grid fields (0.174 mm^2^ per animal) were evaluated.

#### Tubulointerstitial damage

Multiple longitudinal sections were examined and graded for the extent of cortical and outer medullae involvement of tubule interstitial damage on a scale of 0 to 4: 0) normal; 1) involvement of less than 10% of the cortex and outer medullae; 2) involvement of up to 25% of the cortex and outer medullae; 3) involvement of 50 to 75% of the cortex and outer medullae; and 4) extensive damage involving more than 75% of the cortex and outer medullae. Tubulointerstitial damage was defined as tubular necrosis, presence of an inflammatory cell infiltrate, tubular lumen dilatation, or tubular atrophy (23).

### Statistical analysis

The data are reported as means±SE. Two-way analysis of variance (ANOVA) was used to assess the interaction between groups (NAC and ischemia). *Post hoc* Tukey and independent *t*-tests were used for pairwise comparisons of groups. Statistical significance was defined at P<0.05. All statistical analyses were performed with Action Stat (version 3.0; EstatCamp, Brazil).

## Results

### NAC treatment improved renal function in short-term ischemia

Data illustrating the effect of NAC on renal function after I/R injury are shown in Table 1. Rats submitted to short- and long-term renal ischemia showed an increase in serum Cr and a decrease in Cr clearance. NAC decreased serum Cr and improved Cr clearance in short-term ischemia, whereas it increased serum Cr and decreased Cr clearance in the long-term ischemia group. The FENa levels in ISC 30 and ISC 30 + NAC were not statistically significant compared to the control groups, whereas in ISC 45 and ISC 45 + NAC groups, the FENa levels increased significantly (P<0.001) compared to the short-term and control groups.

**Table 1:** Effect of n-acetylcysteine (NAC) treatment on renal function after renal ischemia reperfusion injury.

Groups	Serum creatinine (mg/dL)	Creatinine clearance (mL/min)	FENa (%)
SHAM	0.3±0.1	0.97±0.02	0.29±0.30
SHAM + NAC	0.2±0.1	0.93±0.04	0.28±0.07
ISC 30	2.1±0.6^ab^	0.12±0.01^ab^	0.80±0.46
ISC 30 + NAC	0.6±0.2^c^	0.57±0.08^abc^	0.73±0.22
ISC 45	2.9±0.5^abc^	0.04±0.01^abcd^	7.50±1.01^abcd^
ISC 45 + NAC	3.9±0.4^abcde^	0.02±0.01^abcd^	11.52±1.91^abcde^

Data are reported as means±SE. The creatinine clearance was adjusted by 100 g/rat weight. FENa: fractional excretion of sodium; ISC 30: ischemia for 30 min; ISC 45: ischemia for 45 min. ^a^P<0.001 *vs* SHAM; ^b^P<0.001 *vs* SHAM + NAC; ^c^P<0.001 *vs* ISC 30; ^d^P<0.001 *vs* ISC 30 + NAC; ^e^P<0.001 *vs* ISC 45 (ANOVA).

### NAC increased RBF after renal I/R in short-term ischemia, but did not change in long-term ischemia

As shown in Table 2, no difference in HR was detected among the groups. An elevation on MAP was observed in the ISC 45 and ISC + NAC groups. Treatment with NAC significantly increased RBF and decreased RVR in short-term ischemia, whereas in long-term ischemia, NAC did not improve these parameters.

**Table 2:** Effect of n-acetylcysteine (NAC) treatment on hemodynamic parameters after renal ischemia reperfusion injury.

Groups	HR (bpm)	MAP (mmHg)	RBF (mL/min)	RVR mmHg·mL–1·min–1
SHAM	455±38	113±6	10.4±1.4	11.3±1.5
SHAM + NAC	466±56	113±7	9.1±2.5	12.9±2.5
ISC 30	483±31	104±2	4.0±0.6^ab^	27.4±1.6^ab^
ISC 30 + NAC	413±67	120±9^c^	7.1±0.5^ac^	15.7±1.1^c^
ISC 45	459±14	107±3^d^	2.3±0.2^abd^	46.0±2.6^abcd^
ISC 45 + NAC	431±57	126±6^abce^	2.9±0.7^abd^	49.7±7.4^abcd^

Data are reported as means±SE. HR: heart rate; MAP: mean arterial pressure; RBF: renal blood flow; RVR: renal vascular resistance; ISC 30: ischemia for 30 min; ISC 45: ischemia for 45 min. ^a^P<0.001 *vs* SHAM; ^b^P<0.001 *vs* SHAM + NAC; ^c^P<0.001 *vs* ISC 30; ^d^P<0.001 *vs* ISC 30 + NAC; ^e^P<0.001 *vs* ISC 45 (ANOVA).

### NAC treatment reduced oxidative stress in short-term ischemia, but not in long-term ischemia

The effects of NAC treatment on the redox status of ischemic AKI are summarized in Tables 3 and 4. Treatment with NAC decreased urinary peroxides, nitrite excretion, and TBARS, with an increased antioxidant capacity, confirmed by GSH-Px activity in short-term ischemia. NAC exposure in long-term ischemia elevated urinary TBARS compared to the treatment with NAC in short-term ischemia, despite the improvement in the GSH-Px activity in renal tissue.

**Table 3:** Effect of n-acetylcysteine (NAC) treatment on reactive oxygen and nitrogen species metabolites after renal ischemia reperfusion injury.

Groups	Urinary peroxides (nmol/g urinary Cr)	Nitric oxide (nmol/g urinary Cr)	TBARS (nmol/g urinary Cr)
SHAM	1.7±0.1	30.2±2.2	54.4±15.6
SHAM + NAC	1.4±0.4	29.0±5.2	69.8±5.5
ISC 30	5.9±0.2^ab^	76.6±6.6^ab^	98.7±12.5^ab^
ISC 30 + NAC	2.4±0.4^c^	31.1±2.4^c^	58.1±16.4^c^
ISC 45	9.7±1.8^abcd^	76.2±14.5^abd^	83.9±8.6^ad^
ISC 45 + NAC	7.8±1.6^abcde^	82.9±5.0^abd^	104.8±13.7^abd^

Data are reported as means±SE. Cr: creatinine; TBARS: thiobarbituric acid-reactive substances; ISC 30: ischemia for 30 min; ISC 45: ischemia for 45 min. ^a^P<0.05 *vs* SHAM; ^b^P<0.001 *vs* SHAM + NAC; ^c^P<0.05 *vs* ISC 30; ^d^P<0.05 *vs* ISC 30 + NAC; ^e^P<0.05 *vs* ISC 45 (ANOVA).

**Table 4:** Effect of n-acetylcysteine (NAC) treatment on antioxidant capacity after renal ischemia reperfusion injury.

Groups	Thiols (nmol/mg total protein)	GSH-Px activity (U/g of renal tissue)
SHAM	204.3±25.3	12.1±4.9
SHAM + NAC	200.8±12.5	11.4±1.6
ISC 30	109.3±9.5^ab^	7.3±1.7
ISC 30 + NAC	95.6±3.7^ab^	14.5±3.0^c^
ISC 45	109.2±35.1^ab^	8.3±1.3^d^
ISC 45 + NAC	103.7±20.0^ab^	13.2±2.8^c^

Data are reported as means±SE. GSH-Px: glutathione peroxidase; ISC 30: ischemia for 30 min; ISC 45: ischemia for 45 min. ^a^P<0.05 *vs* SHAM; ^b^P<0.001 *vs* SHAM + NAC; ^c^P<0.05 *vs* ISC 30; ^d^P<0.05 *vs* ISC 30 + NAC (ANOVA).

### NAC attenuated renal tubular injury in the short-term I/R model of AKI

**Figure 1:**
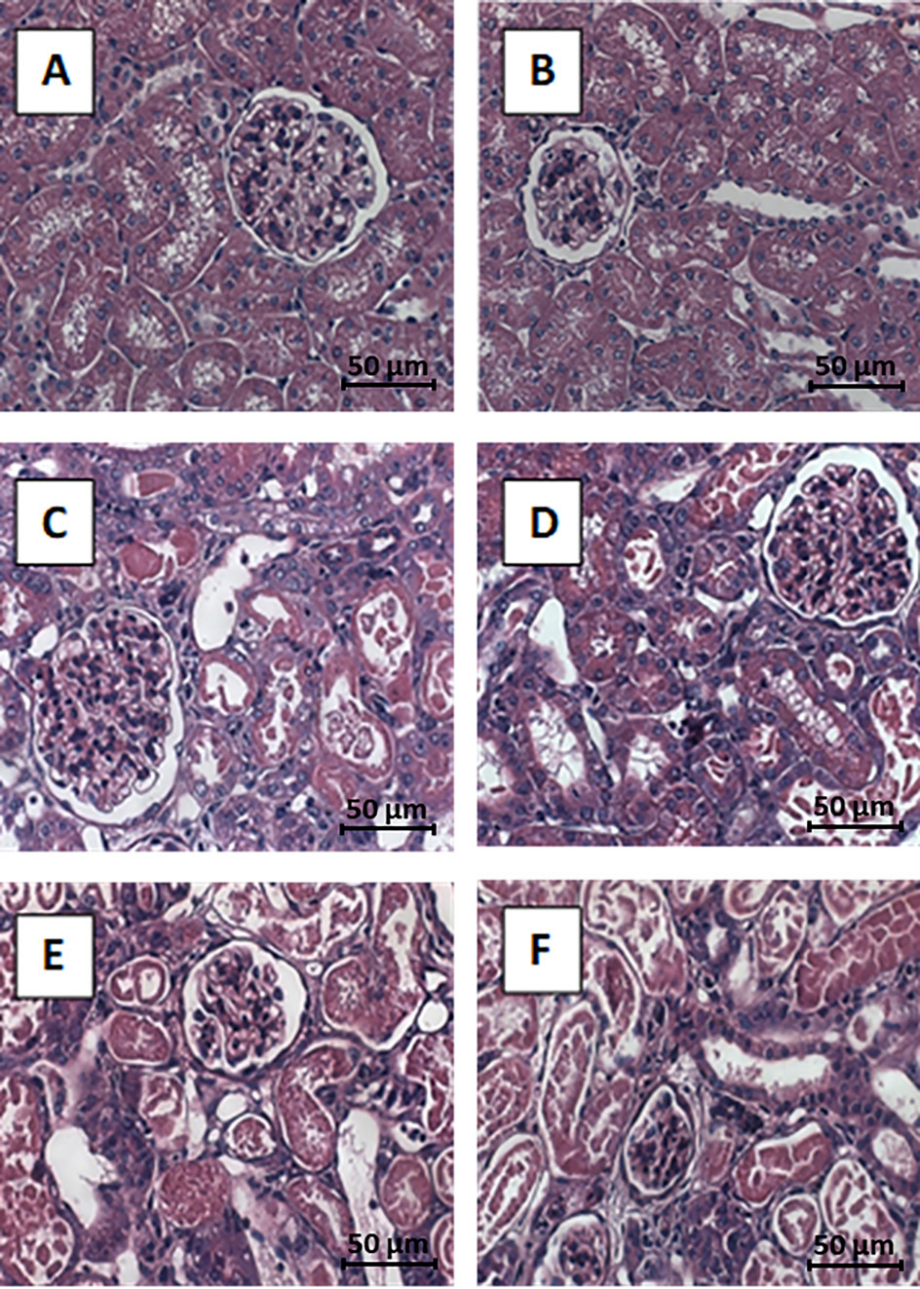
Effect of n-acetylcysteine (NAC) on renal ischemia reperfusion (I/R) injury. Sham (**A**) and SHAM + NAC (**B**) kidneys presented normal histology. Ischemia (ISC) 30 min (**C**), ISC 30 + NAC (**D**), ISC 45 min (**E**), and ISC 45 + NAC (**F**) displayed tubulointerstitial damage as defined by tubular necrosis, inflammatory cell infiltration, tubular lumen dilatation, or tubular atrophy. HE staining (scale bar 50 μm).

Figure 1 shows the representative images of renal histological analysis. After I/R, kidneys presented tubulointerstitial injury characterized by edema and diffuse inflammatory interstitial infiltration, flattening of tubular cells, areas of denude basement membrane, and tubular necrosis in the cortex and outer medullae.

**Figure 2:**
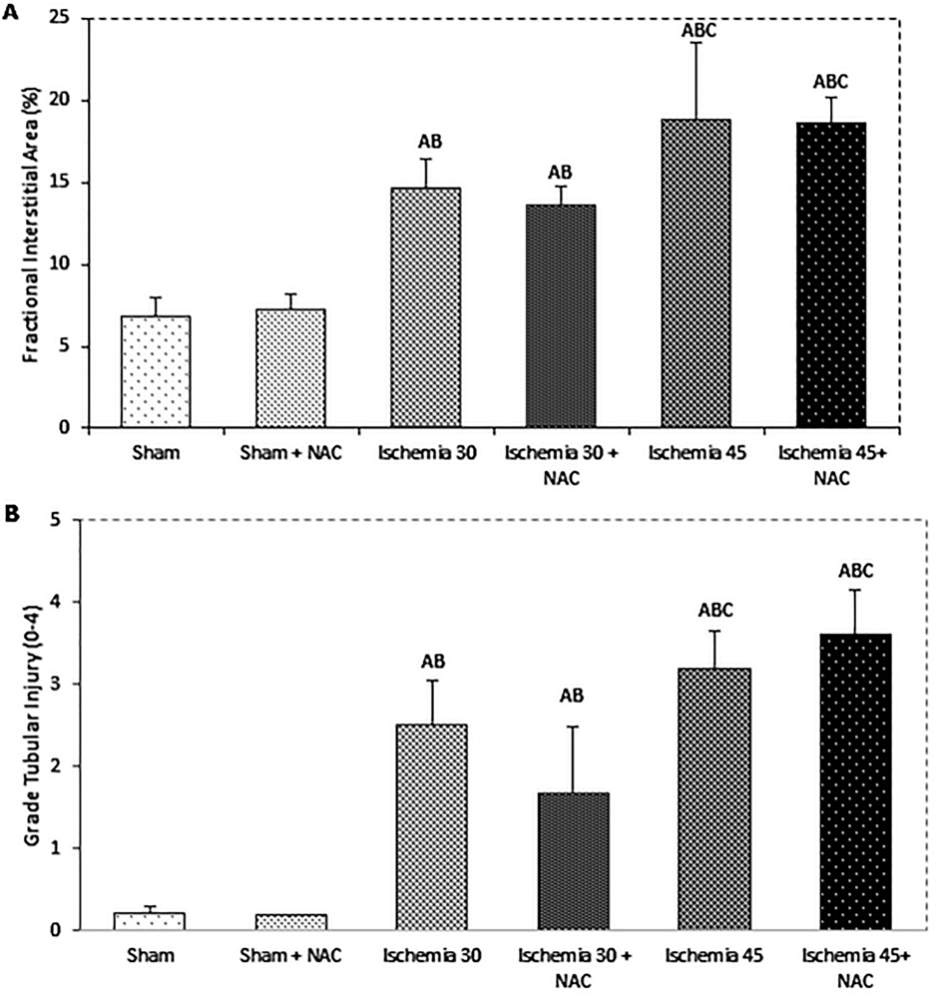
Effect of n-acetylcysteine (NAC) on renal histological analysis. Data are reported as means±SE. ^A^P<0.001 *vs* SHAM; ^B^P<0.001 *vs* SHAM + NAC; ^C^P<0.001 *vs* Ischemia 30 min (ANOVA).

Figure 2A shows that short- (14.6±0.4%) and long-term (18.8±2.1%) ischemic kidneys demonstrated an increase in FIA compared to the control groups (P<0.001). Additionally, long-term renal ischemic groups exhibited a statistically significant increase in FIA compared to short-term groups (P<0.001). No effect of NAC was observed on FIA.

Tubulointerstitial damage is shown in Figure 2B. The grades of tubular injury in the ISC 30 group (2.5±0.2) demonstrated damage of up to 25% of the cortical and outer medullae area, and the ISC 45 group (3.2±0.2) exhibited progression of tubular injury up 50 to 75% (P<0.001). There was no effect of NAC treatment on short- and long-term ischemia.

## Discussion

AKI is associated with high morbidity, increased costs, and mortality (1-3). Treatments of AKI are needed to reduce the high morbidity and mortality, improve recovery of renal function, and reduce progression to chronic kidney disease and end-stage renal disease (24). Also, ischemic AKI continues to be a significant challenge in maintaining renal function and preserving graft viability.

Despite the improvements in clinical settings, renal function after AKI remains far from ideal and without any therapeutic option. There are certainly several reasons for this: complex pathogenesis and multifactorial etiology of AKI, timely prophylactic or early pharmacological treatments that can promote recovery are not applied, and a complicated clinical course in hospital settings, such as ischemic kidney injury due to surgery, hemorrhage, sepsis, trauma, or toxic responses to medications. Our results are an advance to the issue in demonstrating that NAC improved renal function by reducing oxidative injury and attenuating tubule interstitial injury in short-term I/R injury, while it was not effective in the long-term model. This different pattern of protection could be due to the unknown temporal course of this oxidative imbalance (25). Previous studies on I/R injury with NAC treatment demonstrate a decrease in oxidative stress injury, renal tubular apoptosis, and systemic or local inflammation (24), but it is also known that most antioxidant strategies developed to treat patients with AKI have failed (26-28).

Maybe the increase in damage observed in the 15 min longer ischemia suggests the ineffectiveness of the protective action of antioxidant substances (29). NAC protected renal function when administered with short ischemia but did not have any effect after a longer ischemia period, highlighting the existence of a “therapeutic window”. The 15-min difference between the two experimental periods caused an exacerbation of oxidative stress and imbalance between the production of ROS and the ability of enzymes to counteract these oxidative radicals, leading to significant and irreversible tissue damage. The persistent mitochondrial dysfunction within damaged tubules after long I/R may lead to chronic deficiencies in cell and organ function and contribute to the sustained injury causing renal failure linked to tubular epithelial cell proliferation with irreversible acute tubular necrosis (30,31). Additionally, as a consequence of oxygen interacting with dysfunctional mitochondria, a burst of superoxide production upon reperfusion and elevation in ROS production occurs (32). This ROS leakage after reperfusion could not be buffered by NAC resulting in the ineffectiveness of this treatment after a long time of ischemia.

Renal ischemia results from the restriction of blood supply, which compromises oxygen and nutrient delivery. Kidneys become fully anoxic and nonfunctioning, causing microvascular dysfunction, decreased glomerular filtration rate (GFR), and impairment of tubular transport activity (2,3). Renal microcirculation plays critical roles in the pathology of AKI. Alteration in RBF has a negative impact in kidney function in both experimental and clinical settings, including impaired capillary perfusion and endothelial barrier failure (obstruction, leaky and broken capillaries). These manifestations are emerging as determinants of the severity of tissue injury outcomes, which depend on the duration of the ischemic reperfusion period (3-8,24). Restoration of blood flow and reoxygenation after ischemia are also involved with injuries. Renal injury gradually worsens upon reperfusion by the activation of inflammatory response and excessive amounts of ROS and RNS (3,6).

This study confirmed the gradual glomerular and tubular dysfunction relative to the ischemic time period. Short-term (30 min) ischemia was characterized by reduced RBF and reduced GFR with elevated ROS and RNS. With longer ischemic time, AKI becomes more severe. Long-term ischemia (45 min of renal I/R injury) demonstrated progressive and consistent renal dysfunction, reduced RBF and increased oxidative injury, increased tubular interstitial area, presence of tubular necrosis, intraluminal casts formation, and areas of denuded basement membrane in the renal cortex, suggesting that, after 45 min of ischemia, renal dysfunction evolved into tubular injury, with decreased GFR and sodium reabsorption (3).

Despite the frequent use of NAC in different models of AKI, the present study demonstrated that, in severe renal ischemia (45 min), NAC is not an effective treatment. The administration of NAC in long-term renal ischemia presented no significant effect on renal function and did not attenuate the oxidant injury at the dose used, despite the increase of antioxidant capacity by GSH on renal tissue. The non-effectiveness of NAC can be justified by the severity of the kidney dysfunction. An experimental model showed similar results after intravenous bolus of NAC prior to ischemia followed by a continuous infusion, where NAC did not improve kidney function during the first 80 min after I/R (33). These could be explained by the release of urinary nitrite especially in the long-term ischemia. Urinary nitrite is a metabolite of nitric oxide (NO), an endothelial cell product that plays an important role in blood circulation (6,34). NO produced in the renal proximal tubules in response to ischemic injury is mediated by iNOS and reacts with the radical O_2_^–^ leading to the formation of peroxynitrite (ONOO-). The ONOO- acts as a strong oxidant in renal homeostasis and adds to the endothelial cell injury and tubular cell damage (34,35).

NAC is a small molecule containing a thiol group with ready access to intracellular compartments, acting as a precursor of L-cysteine, which promotes GSH synthesis, directly involved in the removal of toxic aldehydes and peroxides (10,12). Studies have shown that NAC provides protection for kidney tissue against oxidative injury partly by a direct reaction with OH^–^ and H_2_O_2_, and also by indirectly facilitating glutathione biosynthesis, which represents a major cellular defense system against oxidative injury (12,36). Thus, NAC can reduce lipid peroxidation of cellular membranes in renal cells, which improves membrane fluidity, permeability, and transport of ions and solutes, but its optimal time to be used is still unknown (7,37).

In this study, the dose of 150 mg/kg was chosen as previously described (15). Several doses of NAC from around 0.204 up to 2 g/kg per day in *in vivo* studies have been shown to remove oxidation of cellular sulfhydryl groups in I/R models (5). On the other hand, administration of high doses of NAC can have a pro-oxidant effect, possibly mediated by the reduction of transition metals and enhancement of the Fenton reaction. Recently, it has been reported that long-term NAC treatment is not effective and is deleterious for function recovery after kidney injury (10,26).

The potential of NAC as a therapeutic drug in different diseases with ROS and inflammatory response was also demonstrated, but further studies are required to identify therapeutic protocols that take into consideration that tissue responses depend on the duration and the magnitude of the injury. This study has shown that NAC can reduce cellular dysfunction or death only at the onset of reperfusion occurring before irreversible ischemia damage within a therapeutic window (4,38). Also, results suggest that the irreversible ischemia damage must have occurred in the 15 min following the short-term ischemia.

In summary, this study has demonstrated that the duration of the ischemia is probably the main determinant factor to the progression and severity of renal dysfunction. The earlier the identification of the lesion in clinical practice, the greater the chances of recovery of the injured renal parenchyma. Therefore, these observations suggested that the clinical efficacy of NAC could be very useful in preventive protocols for patients with chronic diseases or diabetes that present alterations in the delicate redox balance or in microvascular reactivity. Nevertheless, further studies are necessary to understand the molecular properties of NAC at different levels of severity of AKI associated with a critical cellular oxidative stress.
